# A model‐based method for reporting mammographic diagnostic reference levels for any compressed breast thickness: A refined reporting approach

**DOI:** 10.1002/acm2.70206

**Published:** 2025-08-21

**Authors:** Ahmad A. Alhulail, Salman M Albeshan, Maha M. Almuqbil

**Affiliations:** ^1^ Department of Radiology and Medical Imaging, College of Applied Medical Sciences Prince Sattam bin Abdulaziz University Al‐Kharj Saudi Arabia; ^2^ Department of Radiological Sciences, College of Applied Medical Sciences King Saud University Riyadh Saudi Arabia; ^3^ Cancer Control Program Ministry of Health Riyadh Saudi Arabia

**Keywords:** average glandular dose, compressed breast thickness, diagnostic reference level

## Abstract

**Background:**

Mammography is a critical tool for early breast cancer detection, but its use of ionizing radiation necessitates careful monitoring and optimization of patient exposure to ensure safety. Conventional methods for reporting diagnostic reference levels (DRLs) rely on wide compressed breast thickness (CBT) ranges, which lack the precision to account for individual variations, limiting their effectiveness in optimizing mammographic radiation doses.

**Purpose:**

To develop an equation‐based approach that provides a DRL for any given CBT.

**Methods:**

The 75th percentile of median average glandular dose (AGD) values from nine centers (a total of 187,704 mammograms) was employed for the DRL estimation using three approaches: (1) estimating a DRL for a CBT range assumed to represent typical women in the population (simplest/common approach), (2) reporting DRLs per different 10‐mm CBT ranges (improved approach), and (3) as a DRL equation (the proposed approach), which was generated from fitting the values of the estimated DRLs versus each corresponding CBT. The differences in these approaches’ results were compared.

**Results:**

For our population, the curve fitting of the DRLs versus their corresponding CBTs resulted in a bi‐exponential equation with high‐fitting reliability (*R* ^2^ > 0.98). The equation approach provides continuous DRL values to serve any given CBT. The difference in the estimated DRLs by the equation approach and the improved range‐based reporting approach can range between several percentages to more than 35% and can exceed that to more than 175% when compared with the estimated simplest approach's DRL.

**Conclusions:**

The proposed DRL equation approach is reliable and can be used to provide more precise results for any given CBT value rather than the conventional range‐based reporting approaches. Vendors can adopt the proposed approach by integrating an option to input DRL equations to automate the optimization of mammographic dose.

## 1 INTRODUCTION

Screening women's breasts by mammography is a well‐established modality for the early detection of abnormal tissue changes.^1^ However, mammography uses ionizing radiation, and the glandular tissue of the breast is considered one of the most sensitive tissues compared with other body tissues.^2^ Indeed, exposing women's breasts to ionizing radiation was shown to be associated with an increased risk of breast cancer. According to the National Health Service Breast Screening Programme, the risk of radiation‐induced cancer is 1 in 20,000 per mammographic screening (i.e., for two views).^2^ However, the screening detects approximately 170 cancers for every radiation‐induced cancer, and the mortality benefit exceeds the radiation risk by roughly 100:1.^2^ Therefore, mammographic radiation doses must be carefully monitored and optimized to preserve this highly favorable benefit‐risk balance.

The mammographic radiation dose is affected by breast composition and thickness.^3^ However, these factors might vary with populations, as each nation's population is unique and has different demographics. Nevertheless, breast cancer may behave differently from one population to another. For instance, breast cancer affects Saudi women at a younger age (approximately 50 years) compared to 62 years in the United States.^4^ Therefore, aiming to improve breast cancer outcomes and prognosis, breast cancer screening programs in different nations have been implemented. Parallelly, each nation started to drive and report their mammographic diagnostic reference levels (DRLs) for their population to maintain mammographic radiation exposure under control.^5^, ^6^, ^7^, ^8^, ^9^ The DRL is a critical tool for radiation dose monitoring and optimization that is recommended by the International Commission on Radiological Protection (ICRP) and the International Atomic Energy Agency (IAEA).^10^, ^11^ ICRP defines DRLs as: “A form of investigation level used as a tool to aid optimization of protection in the medical exposure of patients for diagnostic and interventional procedures”.^10^

In mammography, the average glandular dose (AGD) is considered the most relevant measure of radiation doses delivered to the breast by several international organizations,^12^ such as the ICRP and IAEA.^10^, ^11^ National DRL values are determined using the 75th percentile of median AGD values collected from a sample of representative centers.^10^ The delivered AGDs in mammographic centers are monitored by comparing them to the established national DRLs as a process of controlling mammographic doses.^11^

The conventional and simplest method of reporting the DRL involves targeting a single 10‐mm range of compressed breast thickness (CBT) that is assumed to represent a standard patient.^10^ In the ICRP Publication 135 (point 372), using the 50  ±  5 mm thickness range was suggested for the simplest approach.^10^ However, this approach poses challenges since the size of standard patients and the typical range of CBT might vary among countries. For instance, previous DRL studies on different populations have suggested different CBT ranges of 45 ± 5,^13^ 55 ± 5,^5^ and 60 ± 5 mm.^6^, ^7^ Additionally, while the one CBT range can roughly represent the majority, it might not be applicable to the minorities within the population, who also deserve consideration for dose monitoring. An improved approach to reporting DRL by reporting multiple DRLs for different 10‐mm CBT range increments (covering the CBTs between 20–110 mm) was introduced and implemented in recent works.^5^, ^6^, ^8^, ^9^ This improvement enhances the precision, but 10 mm may still be considered a wide range and further refinement to provide DRL values for different breast thicknesses is recommended, as highlighted within the ICRP Publication 135 in points 157 and 372,^10^ as well as in an earlier work that focused on DRL limtations.^14^

To address the limitation in the preceding paragraph, we propose a comprehensive, equation‐based method that delivers precise, continuous DRL values for any CBT, thereby enhancing DRL reporting and optimizing mammographic dose. By adopting this method, we can achieve a better evaluation of radiation dose and enhance the overall effectiveness of mammographic protocols.

## 2 MATERIALS AND METHODS

### 2.1 Subjects and data collection

Ethical approval was granted by the research ethics committee at the corresponding author's institute before starting the project. In this retrospective analysis, data from 46,947 women's examinations (187788 mammogram images) were gathered, which were acquired by nine centers between 2012 and 2021 as part of the Saudi breast screening program. Related data in Table 1, including the exposure factors, patient, machine specifications, and radiation dose, were extracted from the image DICOM header. This information was automatically exported to a CSV file via MATLAB (MathWorks, Natick, MA) coding.

**TABLE 1:** Data extracted from the image DICOM header.

1. Patient's age
2. Exposure (mAs)
3. Exposure control mode
4. Tube voltage (kVp)
5. CBT
6. Compression force
7. Implant present
8. Image laterality
9. View
10. Anode target material
11. Filter material
12. Manufacturer's model name
13. Manufacturer
14. Organ Dose (The AGD measured by the machine)

Abbreviations: AGD, Average Glandular Dose; CBT, Compressed breast thickness; DICOM, Digital Imaging and Communication in Medicine.

Only data that includes the standard mammographic projections (left and right mediolateral oblique (MLO), and left and right craniocaudal (CC)) were included. Further, any examination that included a mammogram view acquired with an extreme value of compression force (greater than 200 N or equal to 0 N) was removed. Similarly, examinations with tube current between 0 and 10 mAs, or tube voltage equal to 0 Kilovoltage Peak (kVp) were dealt with as erroneous data and were excluded as well. Data from CBT more than 100 mm were also excluded because there were a few, which may degrade the reliability of their results. The number of examinations, subjects' characteristics, and exposure parameters are summarized in Table 2.

**TABLE 2:** Number of examinations (n), subjects' characteristics, and exposure parameters.

Center	*n*	Age [y]	CBT [mm]	mAs	kVp	CF [N]
		Mean (SD; range)				
1	38028	51 (8; 30–90)	57 (12; 13–100)	82 (22; 11–335)	29 (1; 25–40)	136 (36; 30–200)
2	31092	50 (8; 30–89)	57 (12; 17–100)	66 (21; 11–286)	29 (1; 25–35)	148 (35; 20–200)
3	24000	50 (8; 30–90)	59 (11; 14–100)	57 (18; 15–335)	29 (1; 23–31)	145 (36; 20–200)
4	60899	49 (8; 30–88)	59 (11; 15–100)	66 (13; 18–335)	29 (1; 25–31)	130 (37; 20–200)
5	15584	49 (9; 30–85)	60 (12; 18–100)	53 (15; 27–238)	29 (1; 24–32)	143 (36; 30–200)
6	16321	47 (8; 30–86)	59 (13; 17–100)	143 (40; 14–404)	29 (2; 25–39)	105 (21; 45–196)
7	628	50 (8; 30–70)	48 (11; 15–82)	58 (12; 32–121)	29 (1; 26–33)	141 (45; 47–200)
8	493	51 (8; 34–68)	46 (12; 22–79)	56 (12; 18–102)	29 (1; 26–32)	174 (26; 54–200)
9	660	48 (8; 30–86)	51 (11; 17–87)	60 (14; 21–132)	29 (1; 26–33)	131 (36; 14–199)
All	187704	49.6 (8; 30–90)	58 (12; 13–100)	74 (31; 11–404)	29 (1; 23–40)	135 (37; 14–200)

Abbreviations: CBT, compressed breast thickness; CF, compression force; kVp, kilovoltage peak; mAs, milliampere‐seconds; SD, standard deviation.

### 2.2 DRL calculation

#### 2.2.1 DRL estimation for the typical CBT range (the common/simplest approach)

Here, the DRL was estimated by following the simplest approach suggested in the ICRP Publication 135 (in point 372) by considering only the breasts of 50  ±  5 mm thickness.^10^ Focusing on this CBT range, the 75th percentile of the AGD medians of each center was used to estimate the DRL for each mammographic view.

#### 2.2.2 DRL estimation per CBT ranges (improved approach)

In the improved approach, the 75th percentile of center medians was also used, but now to calculate a DRL for each 10‐mm CBT increments between 20 to 100 mm.

#### 2.2.3 DRL equation for any given CBT (proposed approach)

In order to start a continuous DRL estimation and go beyond the wide CBT range/s, a DRL equation was derived to allow the estimation of a refined DRL value for each distinct CBT. First, the 75th percentiles found at the center of each 10‐mm‐CBT increment were plotted as a function of their corresponding CBT. The plot data were then fitted with different models before using the best representative model based on the coefficient of determination (R^2^), adjusted R^2^, sum of squared errors (SSE), and root mean square error (RMSE) values. The tested fitting models were linear, power (one and two terms), and (mono‐ and bi‐) exponential models. The Trust‐region algorithm was employed when the nonlinear models were used. No starting or extra constraint values were applied during the fitting processes. This was done for the CC and MLO data separately to generate an equation for each mammographic view.

### 2.3 Effect of mammography machine technology on the DRL equation estimation

The equation approach was performed separately for data acquired by the different mammography machines (Hologic Lorad Selenia, GE Senographe Essential, and Fujifilm FDR‐3000AWS) to investigate the potential effect of their varying technology on the DRL equation estimation.

## 3 RESULTS

### 3.1 DRL estimation for the typical CBT range (the common/simplest approach)

Limiting the calculation to the 50 ± 5 mm CBT range, the DRL was found to be 1.49 mGy for the CC view and 1.69 mGy for the MLO view.

### 3.2 DRL estimation per CBT ranges (improved approach)

**FIGURE 1:**
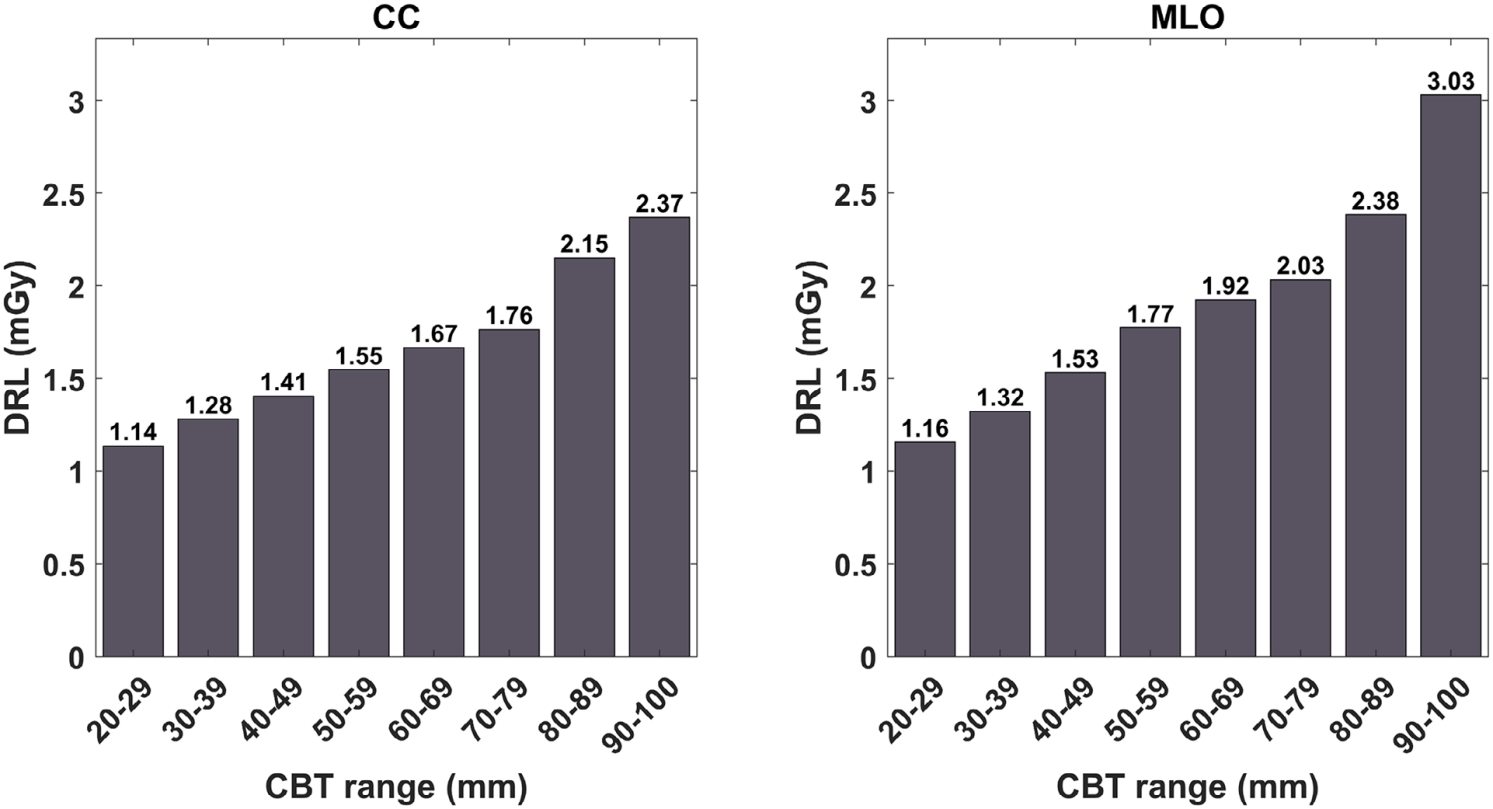
The DRL values calculated per 10‐mm CBT ranges for the CC and MLO views separately. CC, craniocaudal; CBT, compressed breast thickness; DRL, diagnostic reference level; MLO, mediolateral oblique.

Figure 1 shows the DRL values corresponding to each 10‐mm‐CBT range from 20 to 110 mm for each mammographic view.

### 3.3 DRL equation for any given CBT (proposed approach)

The goodness‑of‑fit results of DRL data for different fitting models were summarized in Table 3 (for the CC view) and Table 4 (for the MLO view). The results were sorted in these tables based on their most representative model (models with minimal SSE listed first). Based on goodness‐of‐fit metrics, the bi‐exponential model was the most representative model for combined data from all machines for both CC and MLO views.

**TABLE 3:** Results of evaluating the fitting models for the CC view DRL curve data (models with minimal SSE listed first as more representative models).

	Fujifilm				
Fitting model:	Biexponential	2‐terms power	Mono‐exponential	Linear	1‐term power
R^2^	0.993	0.988	0.958	0.929	0.881
Adjusted R^2^	0.983	0.979	0.948	0.911	0.851
SSE	0.001	0.002	0.005	0.009	0.015
RMSE	0.021	0.023	0.037	0.048	0.062
	**Hologic**				
**Fitting model**:	**Biexponential**	**Mono‐exponential**	**2‐terms power**	**Linear**	**1‐term power**
R^2^	0.994	0.993	0.992	0.983	0.962
Adjusted R^2^	0.989	0.992	0.988	0.980	0.956
SSE	0.007	0.008	0.010	0.020	0.044
RMSE	0.043	0.037	0.044	0.058	0.086
	**GE**				
**Fitting model**:	**Biexponential**	**2‐terms power**	**Mono‐exponential**	**Linear**	**1‐term power**
R^2^	0.992	0.943	0.928	0.905	0.864
Adjusted R^2^	0.986	0.920	0.916	0.889	0.841
SSE	0.004	0.029	0.036	0.048	0.068
RMSE	0.032	0.076	0.077	0.089	0.107
	**Combined data from all machines**				
**Fitting model**:	**Biexponential**	**2‐terms power**	**Mono‐exponential**	**Linear**	**1‐term power**
R^2^	0.988	0.984	0.981	0.957	0.928
Adjusted R^2^	0.979	0.978	0.978	0.950	0.916
SSE	0.015	0.020	0.024	0.054	0.090
RMSE	0.061	0.063	0.063	0.095	0.122

Abbreviations: CC, craniocaudal; R^2^, coefficient of determination; RMSE, root mean square error; SSE, sum of squared errors.

**TABLE 4:** Results of evaluating the fitting models for the MLO view DRL curve data (models with minimal SSE listed first as more representative models).

	Fujifilm				
Fitting model:	Biexponential	2‐terms power	Mono‐exponential	Linear	1‐term power
R^2^	0.997	0.996	0.915	0.829	0.801
Adjusted R^2^	0.995	0.994	0.898	0.795	0.761
SSE	0.002	0.003	0.060	0.120	0.140
RMSE	0.024	0.026	0.109	0.155	0.167
	**Hologic**				
**Fitting model**:	**Biexponential**	**Mono‐exponential**	**2‐terms power**	**Linear**	**1‐term power**
R^2^	0.989	0.988	0.987	0.976	0.965
Adjusted R^2^	0.981	0.986	0.981	0.972	0.959
SSE	0.029	0.032	0.036	0.064	0.095
RMSE	0.086	0.073	0.085	0.103	0.126
	**GE**				
**Fitting model**:	**Biexponential**	**Mono‐exponential**	**2‐terms power**	**Linear**	**1‐term power**
R^2^	0.989	0.964	0.964	0.949	0.921
Adjusted R^2^	0.981	0.958	0.949	0.940	0.908
SSE	0.007	0.022	0.023	0.032	0.049
RMSE	0.041	0.061	0.067	0.073	0.090
	**Combined data from all machines**				
**Fitting model**:	**Biexponential**	**2‐terms power**	**Mono‐exponential**	**Linear**	**1‐term power**
R^2^	0.993	0.970	0.968	0.935	0.918
Adjusted R^2^	0.987	0.958	0.963	0.924	0.904
SSE	0.019	0.077	0.082	0.168	0.212
RMSE	0.068	0.124	0.117	0.167	0.188

Abbreviations: DRL, DRL, diagnostic reference level; MLO, mediolateral oblique; R2, coefficient of determination; RMSE, root mean square error; SSE, sum of squared errors.

The DRL reporting equation, derived from the bi‐exponential model (the best‐fitting representation), was implemented to produce continuous DRL estimates as a function of CBT. The CC‐view DRL (DRL_CC_) for any given CBT value can be estimated from the following equation:

(1) $$\begin{array}{cc} & DRL_{CC}\left(mGy\right)\\ & =0.94exp\left(CBT/118.65\right)+7.30\times 10^{-4}exp\left(CBT/15.85\right) \end{array}$$

which showed very high fitting reliability (R^2^ = 0.988).

The MLO‐view DRL (DRL_MLO_) for any given CBT value can be estimated from the following equation:

(2) $$\begin{array}{cc} & DRL_{MLO}\left(mGy\right)\\ & =0.912exp\left(CBT/89.31\right)+1.887\times 10^{-14}exp\left(CBT/3.1\right) \end{array}$$

which also showed very high fitting reliability (R^2^ = 0.993).

### 3.4 Effect of mammography machine technology on the DRL equation estimation

**FIGURE 2:**
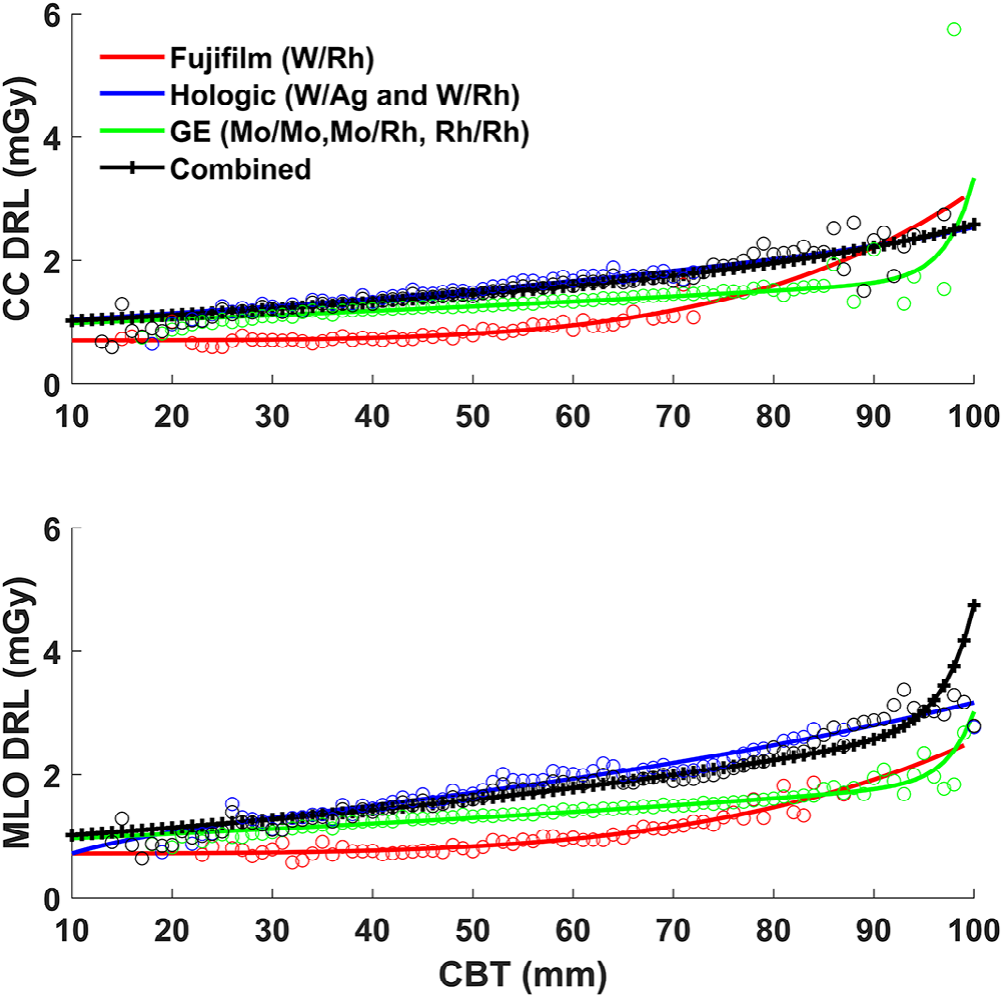
DRL curve for each machine per mammographic view, the CC and MLO. The used target/filter combination is listed for each machine. CC, craniocaudal; DRL, diagnostic reference level; MLO, mediolateral oblique.

Based on the goodness‐of‐fit metrics, the bi‐exponential model followed by the two‐term power model were the most representative models for the CC and MLO data acquired with all machines and for the combined data. The only exceptions were the CC and MLO data from the GE Senographe Essential system and the MLO data from the Hologic Lorad Selenia system, which were best described by the bi‐exponential model followed by the mono‐exponential model (see Tables 3 and 4 and Figure 2).

## 4 DISCUSSION

Aiming to improve the field practice and mammographic dosimetry outcomes, in this work, we suggested a new approach to report the DRL through an equation that covers any given CBT in a more precise way. To show the importance of the proposed method, we compared the different DRL reporting approaches (the simplest approach with 50 ± 5 mm CBT, per different CBT ranges, and the proposed new approach using a DRL equation for any individual CBT). Each method resulted in a different DRL value, but only one of them is supposed to be adopted to reduce confusion and attain the optimum patient protection. Below, we discussed how the proposed DRL equation method can provide superior results and the advantages of using it.

**FIGURE 3:**
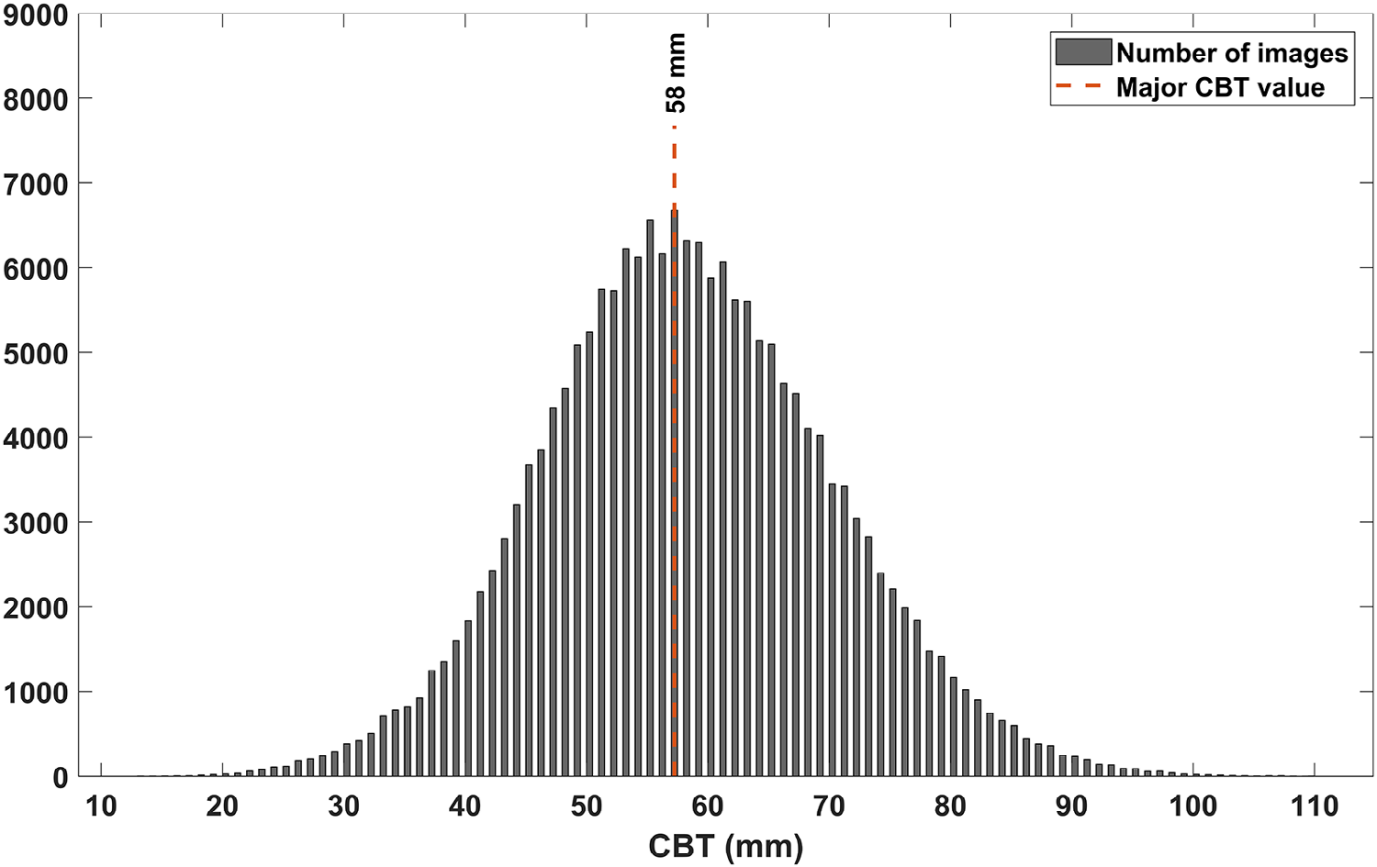
CBT distribution. The 10‐mm CBT range covering the majority of the population is 58 ± 5 mm. CBT, Compressed breast thickness.

The common approach of calculating the DRL based on one wide CBT range, with the assumption that this is the range that represents the typical patient size in the population, might not be reliable. For example, as can be seen in Figure 3, the most common 10‐mm CBT range in our study was found within the 58 ± 5 mm range, which falls beyond the CBT suggested in the ICRP Publication 135 (50 ± 5 mm). In previous DRL reports, the variation among nations in breast size led to using 10‐mm CBT ranges different than 50 ± 5 mm.^5^, ^6^, ^7^, ^13^ Thus, no specific 10‐mm range can be generalized to be used for all national DRLs. Moreover, even if the common CBT range used is representative of the majority, it may not be useful for the minorities, who deserve to be considered too. For instance, the simplest approach (using 50 ± 5 mm CBT) yielded DRLs of 1.49 mGy for CC and 1.69 mGy for MLO, whereas the 10‐mm‐range method at 100 mm CBT produced DRLs of 2.37 mGy (CC) and 3.03 mGy (MLO), representing differences of 59% and 79%, respectively. For the equation approach at 100 mm, the DRLs were found to be 2.58 mGy (CC) and 4.72 mGy (MLO), corresponding to differences of approximately 73% and 179%, respectively, relative to the simplest approach.

**FIGURE 4:**
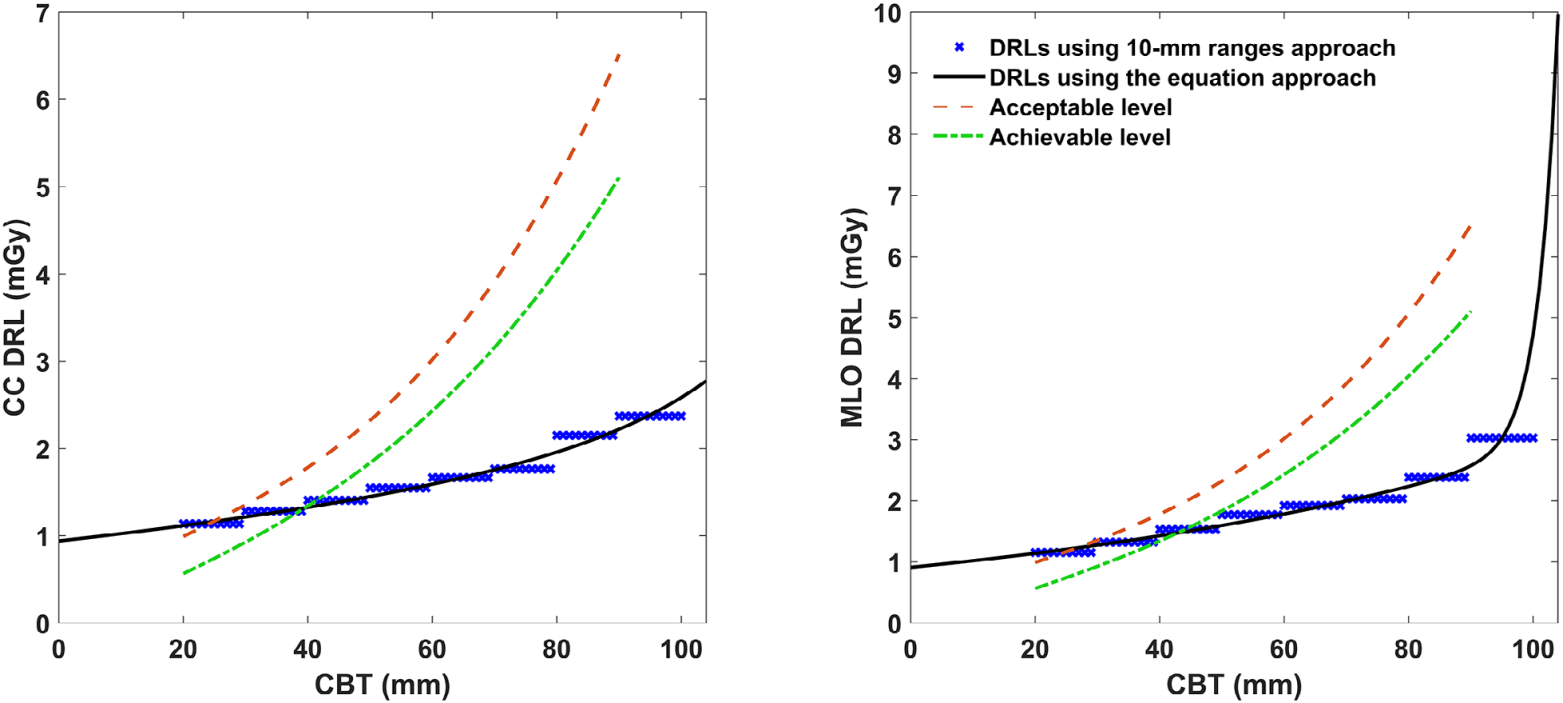
Illustration of the difference between the DRL estimated by the 10‐mm ranges approach and the equation approach. Unlike the ranges approach, the equation approach gives continuous DRL values, allowing more precise DRL estimation for any particular CBT. The variation becomes larger for larger CBTs. The DRL curve provides a reasonable comparison with the acceptable and achievable limits suggested by the IAEA. CBT, compressed breast thickness; DRL, diagnostic reference level; IAEA, International Atomic Energy Agency.

Reporting the DRL per different 10‐mm CBT ranges is an improved approach, which becomes a more acceptable method in the field and has been used in several recent reports.^6^, ^8^, ^9^ We think this CBT multi‐range method is better than the first approach. However, a 10‐mm range is still a wide range of CBT to be assigned one DRL value. Our equation‐based method addresses this by calculating a DRL at each discrete CBT, thereby improving precision. For most CBTs and views, the differences between the 10‐mm range method and the equation approach DRLs are less than 10% (about 0.2 mGy), which might be considered a very limited improvement given the existing very large benefit‐to‐risk ratio of over 100:1 in mammography screening. However, in some cases when considering values away from the 10‐mm ranges’ centers, the variation between the results of the DRLs calculated by these two approaches can exceed 35%, as shown in Figure 4 and the examples in Table 5. A Wilcoxon signed‐rank test was performed to assess the statistical significance of the percent differences between the continuous DRL values estimated by the equation approach and those derived from the 10‐mm range approach, and the differences were found highly significant (P < 0.001). Thus, compared to the range‐based reporting methods, the DRL equation can estimate a more precise value for any individual breast thickness.

**TABLE 5:** Examples of some differences in the DRL results when estimated by the 10‐mm ranges and the equation approaches.

CBT example [mm]	DRL by the 10‐mm range approach [mGy]		DRL by the equation approach [mGy]		Difference^a^	
	**CC**	**MLO**	**CC**	**MLO**	**CC**	**MLO**
50	1.55	1.77	1.45	1.59	6.9%	11%
80	2.15	2.38	1.96	2.24	9.7%	6.3%
100	2.37	3.03	2.58	4.72	8.2%	35.8%

^a^ Difference: The difference between the DRL values estimated by the equation approach and the DRL values falls within the closest 10‐mm CBT range.

Abbreviations: CBT, compressed breast thickness; CC, craniocaudal view; DRL, diagnostic reference level; MLO, mediolateral oblique view.

The refined DRL‐equation approach provides more comprehensive and precise values for each given CBT. If a national DRL was established using this proposed approach, local clinics could optimize their practice by calculating their curves, comparing them to the national one, and trying to optimize their protocols based on it. Additionally, the mammographic DRL curves could be adopted like the CT size‐specific dose (SSD) boundary range. The SSD boundary range was incorporated into protocol definitions and guidelines for radiographers to enhance examination consistency across operations and train them to recognize the causes of overexposures and monitor the change in dose boundary ranges.^11^, ^15^

Moreover, as a step to improve the use of DRL further, manufacturers could integrate a mechanism to add this equation into their automatic exposure control (AEC) systems to automate dose control within the DRL per scanned patient. Thus, we suggest incorporating an option within the mammography machine to plug the derived DRL equations so an optimized and controlled personalized dose can be delivered based on the patient's CBT.

Moreover, as illustrated in Figure 4, using the fitting equation approach can facilitate the comparison with the given achievable and acceptable dosimetry limits that are presentable as curves suggested in the quality documentation of IAEA and European Commission guidelines.^16^, ^17^ For instance, according to Figure 4, considering the range covered by the limits suggested by the guidelines, the Saudi breast screening program DRLs were below the suggested limits for women of CBT ≥ 40 mm. For the women with CBT ≤ 40 mm, the DRLs were above the achievable limits and below the acceptable limits. This indicates a good radiation exposure control practice within the Saudi screening program in general, but efforts to improve the practice for the CBTs ≤ 40 mm are suggested.

When implementing the equation approach, the quality of the data fitting may rely on the sample size; as the sample size increases, a better fitting quality and DRL estimation is expected. In this study, we used a large sample size, which covered various CBT values and provided a high fitting quality (R^2^ ≥ 0.98). Thus, the proposed reporting approach might not provide such a good performance with local DRL estimation if a small sample size is employed. Yet, it is expected to work very well with national DRL reporting studies, or even regional DRL estimations with large sample sizes. This might be considered a limitation of the equation approach. However, the sample size issue is also applicable to the range‐based reporting approaches. When case counts are low, particularly in extreme CBT ranges, mitigation strategies can be implemented, such as using wider (potentially overlapping) CBT windows or combining adjacent extreme intervals. These approaches increase the number of observations per group and thus improve DRL precision. Additionally, the Trust‐region algorithm is a nonlinear fitting method that optimizes parameters within a localized trusted radius, adjusting step sizes by local curvature that prevents overfitting.^18^, ^19^ Thus, it can also be useful when the peripheral data are sparse.

The sample size can affect DRL precision, especially for extreme CBTs, which are usually less frequent. Table S1 in the Supplementary Materials lists the 95 % confidence intervals (CIs) of the median AGD per CBT range, along with the corresponding sample sizes. In general, smaller sample sizes can widen the CI. However, although smaller sample sizes can widen CIs, they do not fully account for the pronounced CI expansion at greater breast thicknesses. Indeed, Table S1 shows that the MLO 90–100 mm range has more than twice the cases (1097) of the 20–29 mm range (433), yet its CI is markedly broader (0.12 mGy versus 0.05 mGy). This suggests that the wider CIs at greater CBTs are not from limited data but from a genuine, steep rise in AGD with larger CBTs, a trend noticed in earlier studies.^8^, ^20^, ^21^, ^22^, ^23^

It should be noted that the equation approach might not always follow one specific model, as it can depend on the employed machine technology. For example, in our study, the first two fitting models have close goodness‐of‐fit coefficients. Even though the bi‐exponential model was always providing the highest fitting quality, the second‐best model was not consistent for all machine data. The bi‐exponential model followed by the 2‐term power model for the data acquired with Fujifilm FDR‐3000AWS, the combined data of all machines, and CC Hologic Lorad Selenia data. However, for the data acquired with GE Senographe Essential and MLO Hologic Lorad Selenia data, the bi‐exponential model followed by the mono‐exponential model (see Table 3 and Figure 2). One of the variations is the difference in the used combination of target/filter materials [FDR‐3000AWS uses one W/Rh combination, Hologic Lorad Selenia uses two combinations (W/Ag and W/Rh), while GE Senographe Essential employs three combinations (Mo/Mo, Mo/Rh, and Rh/Rh)]. In our case, the variation among machine technologies didn't show a difference in the preferred fitting model. However, as it is not fully clear how this and other technical variations may influence the AGD, and eventually the DRL curve trend, a further investigation of their influence is needed. This also suggests a need for efforts by manufacturers to harmonize their technologies' effects on the AGD. Thus, you may need to find the best‐fitting model that represents your data, which may result in a different trendline based on the machine used to collect the mammographs.

The proposed equation‐based method delivers a precise DRL for any CBT, including extreme thicknesses, reducing the unnecessary dose that results from rounding the DRLs over a wide CBT range. This dose reduction gained by this CBT‐specific, continuous estimation aligns with ALARA principles and contributes to minimizing the potential radiation‐induced cancer risk in mammography screening. By employing equation‐based DRLs, facilities can develop tailored DRLs that consider each patient's breast size. In addition to this contribution to the overall improvement of mammography practice, it also emphasizes the commitment to delivering personalized and higher‐quality care that considers the distinctive characteristics of every scanned individual. The individual DRL meets the need expressed in previous publications.^10^, ^14^ When individuals receive optimized AGDs, this would optimize the safety of the entire population. Additionally, having a continuous DRL estimation like the proposed equation‐based method allows easier implementation in automated technologies like the AEC, which operates on a patient‐based dose optimization.

This work makes a step toward improving the overall optimization. However, while this work refines the DRL reporting way, which contributes to a better personalized radiation‐safety care, it only focuses on the dose aspect. The other two essential aspects of dose optimization, image quality, and clinical diagnostic outcomes (particularly, cancer detection rate for mammographic screening) need to be considered as well. After establishing the DRL, periodic monitoring of image quality and clinical outcomes needs to be compared, and if a degradation exists in the outcomes as a result of the dose reduction following the implemented DRLs, the DRLs may need to be updated to higher levels. Good optimization (achieving low but sufficient doses) should preserve or improve detection rates while minimizing radiation risks. By integrating such practices, the establishment of DRLs can more effectively uphold the ALARA principle without compromising diagnostic integrity.

## 5 CONCLUSION

This paper proposes a novel equation‑based approach to report DRL for any individual CBT (rather than relying on a DRL for a wide CBT range). It enhances the concept of DRLs by addressing variations in patient size. The proposed approach is feasible and can provide a comprehensive and more personalized controlling tool, covering a larger population while providing more precise values. Also, it facilitates the comparison with the given achievable and acceptable limits that are presentable as curves for quality assessment. Adopting the proposed method refines the radiation monitoring processes and ultimately enhances the population's radiation safety, as it allows for delivering a more acceptable optimized radiation dose.

## AUTHOR CONTRIBUTIONS

Ahmad A. Alhulail: Conceptualization, Methodology, Formal analysis, Investigation, Visualization, Writing—Original draft. Salman M Albeshan: Data curation, Formal analysis, Investigation, Writing‐ Original draft, Project administration. Maha M. Almuqbil: Resources, Data curation, Writing‐ Reviewing and Editing, Supervision.

## CONFLICT OF INTEREST STATEMENT

The authors declare no conflicts of interest.

## ETHIC STATEMENT

Ethical approval was granted by the Standing Committee of Bioethics Research at Prince Sattam bin Abdulaziz University before starting the project (protocol code SCBR‐029‐2022; date: 29‐May‐2022).

## Supporting information



Supporting information
