# Supplementary material for: A model‐based method for reporting mammographic diagnostic reference levels for any compressed breast thickness: A refined reporting approach
Source: J Appl Clin Med Phys. 2025 Aug 21;26(9):e70206. doi: 10.1002/acm2.70206 (PMC12370403; doi:10.1002/acm2.70206)
Supplement: Supplementary file 1 — Supporting information [file ACM2-26-e70206-s001.docx]

| **TABLE S1** Number of cases and 95% CI widths for median AGD by 10-mm-CBT range per view. | | | | |
| --- | --- | --- | --- | --- |
| **CBT Range [mm]** | **CC cases** | **CC CI width [mGy]** | **MLO cases** | **MLO CI width [mGy]** |
| 20–29 | 927 | 0.04 | 433 | 0.05 |
| 30–39 | 6534 | 0.02 | 2207 | 0.03 |
| 40–49 | 25179 | 0.01 | 8803 | 0.02 |
| 50–59 | 37405 | 0.01 | 23652 | 0.01 |
| 60–69 | 19329 | 0.01 | 31394 | 0.01 |
| 70–79 | 4027 | 0.03 | 20126 | 0.02 |
| 80–89 | 392 | 0.14 | 6174 | 0.04 |
| 90–100 | 25 | 1.15 | 1097 | 0.12 |
| Abbreviations: AGD, Average glandular dose; CBT, compressed breast thickness; CC, craniocaudal view; MLO, mediolateral oblique view; CI, confidence interval | | | | |
